# Phenotypic changes in group B streptococci grown in the presence of the polyols, erythritol, sorbitol and mannitol

**DOI:** 10.1186/s12866-021-02208-z

**Published:** 2021-05-13

**Authors:** Maram Hulbah, Matthew A. Croxen, Gregory J. Tyrrell

**Affiliations:** 1 Division of Diagnostic and Applied Microbiology, Department of Laboratory Medicine and Pathology, University of Alberta, Edmonton, Canada; 2 Department of Clinical Laboratory Sciences, College of Applied Medical Sciences, Taif University, Taif, Saudi Arabia; 3 Alberta Precision Laboratories-Public Health, Edmonton, Alberta T6G 2J2 Canada

**Keywords:** GBS, Polyols, PGK, Virulence, Invasion

## Abstract

**Background:**

Group B streptococci (GBS) are important neonatal bacterial pathogens that can cause severe invasive disease in the newborn. It is thought that in many cases of invasive neonatal GBS disease, the bacteria ascend the vagina into the uterus and infect the amniotic fluid surrounding the fetus. Important constituents of this environment include the polyols or sugar alcohols of which erythritol, sorbitol and mannitol are examples. The aim of our study was to investigate the effect of polyols on GBS grown in media containing these sugar alcohols.

**Results:**

GBS incubated in varying concentrations of polyols (erythritol, sorbitol or mannitol) did not display any significant enhancement or inhibition of bacterial growth. However, growth of GBS in the presence of erythritol significantly increased the surface expression of GBS-PGK (a plasminogen binding protein) 1.25 to 1.5-fold depending on the erythritol concentration and significantly enhanced the survival in human blood 3X to 18X depending on the concentration of polyol used. Interestingly, GBS grown in 1% erythritol significantly increased invasion by the bacteria of HeLa cells (epithelial cell line) (150% vs 100%) however, at higher concentrations (2% or 4% of polyol) the number of CFUs was significantly reduced (55-75% vs 100%) suggesting higher concentrations of polyols may inhibit invasion. Erythritol also increased GBS hemolytic activity as well as enhancing biofilm formation 1.4X to 3.3X depending on the concentration of polyol used.

**Conclusions:**

GBS grown in the presence of polyols alters the bacteria’s phenotype resulting in changes associated with GBS virulence. This effect was greatest for the polyol erythritol.

## Background

Group B Streptococcus (GBS), also known as *Streptococcus agalactiae*, is a Gram-positive encapsulated bacterium which can cause invasive disease in adults as well as pneumonia, sepsis and meningitis in the neonate [1–3]. For neonatal GBS infections, 10% can potentially become lethal, and 25- 35% of surviving infants with meningitis may present with permanent neurological sequelae [1–3]. GBS is also a commensal bacterium of the adult gastrointestinal tract and is present asymptomatically in the vaginal flora of approximately 30% of healthy women [4, 5].

The signals that control the switch from a commensal to a highly virulent bacteria for GBS are not completely clear, although a change in pH from the acidic environment of the vagina to the neutral pH of the blood is thought to be important [6]. One factor that may be associated with this phenotypic change is exposure to polyols. Polyols (e.g. erythritol, sorbitol and mannitol) are sugar alcohols (sugar-free sweeteners), derived naturally from fruits and can be commercially produced [7–9]. Due to their lower caloric content compared to sugars, polyols are added to food, candies and chewing gum [10]. In addition to their advantages as non-caloric sweeteners, they are not broken down by bacteria in the mouth or metabolized to acids, and thus do not contribute to tooth decay [11]. Polyols have been suggested to have preventive properties against caries and several studies have shown that polyols can decrease polysaccharide-producing oral streptococci glass surface adhesion and biofilm formation [12–19].

In contrast to the effect of polyols on oral streptococci, the presence of the polyol erythritol in the placenta of ruminants has been proposed as an explanation for the accumulation of various species of virulent bacteria found at these sites (e.g. *Brucella*)*,* leading to abortion of the fetus [20, 21]. It has been known since the 1960s that the presence of erythritol in the placenta of ruminants can promote replication of *Brucella abortus* to high numbers resulting in abortion [22, 23]. Additional work by Petersen et al., studying the pathogenesis of *Brucella* using a mouse model, demonstrated that *Brucella* can localize to an artificial site containing erythritol within the mouse, resulting in a significant immune response [20]. Microarray analysis of *Brucella* grown in media containing erythritol revealed an up-regulation of several virulence factors including genes related to carbohydrate metabolism such as *pgk* (phosphoglycerate kinase), *gapDH* (glyceraldehyde 3-phosphate dehydrogenase) and *pgm* (phosphoglycerate mutase) [20, 24]. Other bacteria such as *Chlamydia psittaci* and *Burkholderia multivorans* have also been found to be more virulent in the presence of erythritol [21, 25, 26].

Based on these previous observations, we hypothesized that the presence of polyols may influence GBS interactions with its environment changing the phenotype. The objective of this study was to investigate and compare the effect of three different exogenous polyols (erythritol, sorbitol and mannitol) on GBS growth, the surface expression of GBS phosphoglycerate kinase (GBS-PGK), bacterial survival in human blood, the ability of GBS to invade human cells, GBS biofilm formation, and GBS hemolysis.

## Results

### Effect of polyols on the growth of GBS-S19

**Fig. 1:**
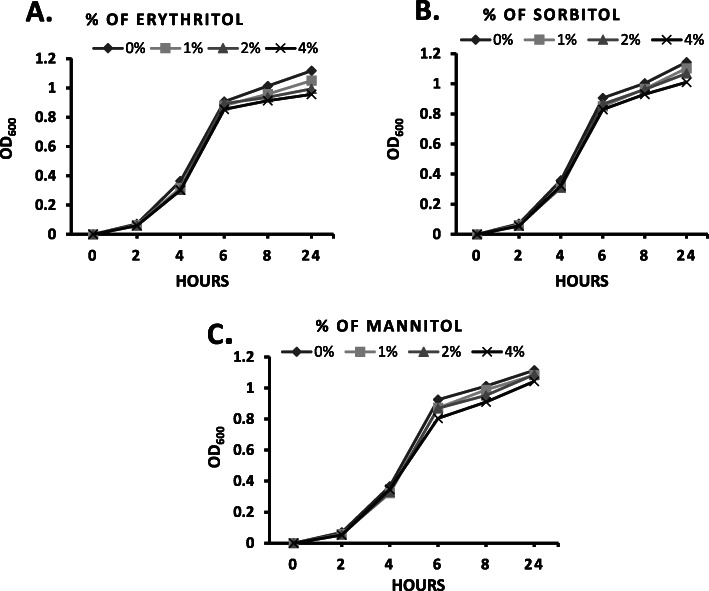
Growth of GBS-S19 in TH broth supplemented with (**a**) erythritol, (**b**) sorbitol or (**c**) mannitol. Growth was assayed by optical density measured at 600 nm for the time points indicated on the x-axis. Data presented is the average OD_600_ of three independent experiments. OD_600_ values were compared with the value obtained from GBS-S19 grown without polyols to determine statistical significance

Polyols were previously shown to have significant effects on the growth of bacteria therefore it was important to determine if this also occurred for GBS [16, 21, 22, 27]. None of the three polyols assayed (erythritol, mannitol or sorbitol) displayed any significant increase or inhibition of GBS growth over a 24-h time period in comparison to control suggesting the polyols do not influence the growth of GBS (Fig.1a, b, c). While growth was unaffected by exposure to polyols, it was unclear if other phenotypic changes occurred upon polyol exposure. To determine this, a series of experiments focused on changes associated with GBS virulence were done.

### Expression of PGK on the surface of GBS-S19 treated with polyols

**Fig. 2:**
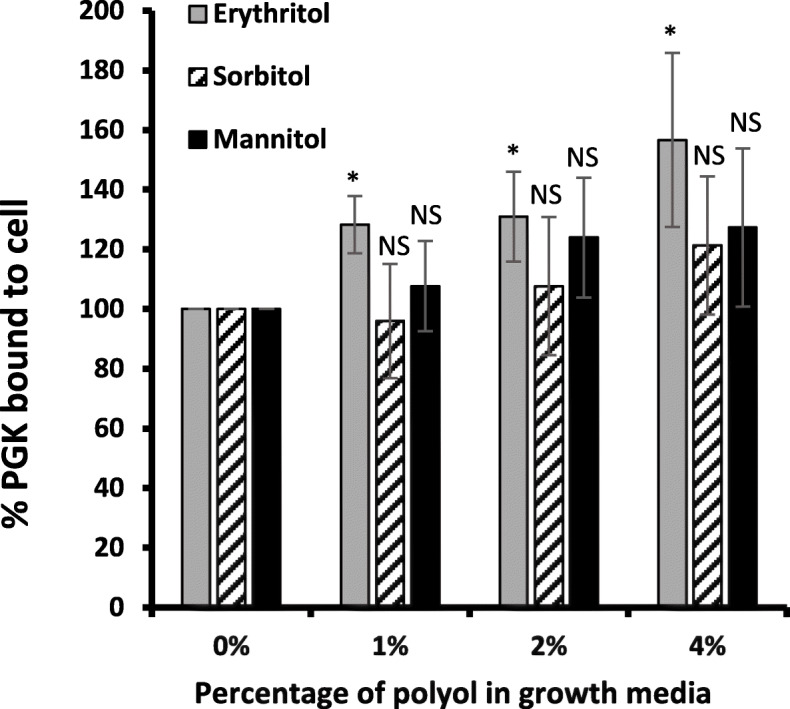
Expression of PGK on the surface of GBS grown in the presence of polyols. GBS-S19 was grown for 20 h in TH broth supplemented with polyols indicated. The quantity of GBS-PGK expressed on the bacterial surface was assayed using polyclonal antibodies against GBS-PGK. Each experiment was performed in triplicate. The bars represent the average A_450_ values compared with GBS grown in THB without addition of polyols (0%). The A_450_ values were compared with the value obtained from (0%) to determine statistical significance; an asterisk (*) indicates statistical significance (*p* < 0.05)

We have previously shown that GBS can express PGK on its surface in addition to intracellular expression [28]. To determine if GBS grown in TH broth supplemented with polyols can affect the surface expression of GBS-PGK, surface expressed GBS-PGK was assayed using antibodies targeting this protein. GBS-S19 cultured in the presence of 1, 2%, or 4% erythritol significantly increased surface expression of GBS-PGK (1.25 to 1.5-fold) compared to growth without erythritol (*p =* 0.003, *p =* 0.012, *p =* 0.014) (Fig. 2). In contrast, GBS-S19 cultured in the presence of the same concentrations of sorbitol or mannitol had no significant effect on the surface expression of GBS-PGK compared with GBS grown without polyols (Fig. 2).

### Effect of polyols on the anti-phagocytic activity of GBS-S19

**Fig. 3:**
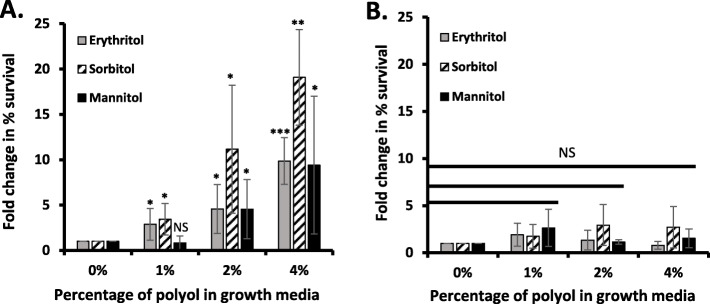
Survival of GBS-S19 in fresh human blood after 20 h growth in the presence of polyols. GBS was grown in TH broth supplemented with polyols and then assayed for the bacteria’s ability to survive in fresh human blood with (**a**) or without (**b**) addition of polyols during incubation. Results are expressed as a fold change in percent of survival in comparison to control GBS-S19 grown in TH broth only. Fold change in percent survival is the number of CFU obtained at the end of the incubation period over the number of CFU obtained at time zero multiplied by 100. Y-axis values in (**a**) and (**b**) are the same in magnitude to allow direct comparison between the two figures. Significance was determined by comparing test values with control (THB with 0% polyols). An asterisk (*) indicates statistical significance (*p* < 0.05), (**) indicates (*p* < 0.01), (***) indicates (*p* < 0.001) and NS indicates not significant

For GBS to cause sepsis and in rare cases meningitis, the bacteria must overcome host defenses including phagocytosis. GBS-S19 first grown in media containing polyols and then incubated in fresh human blood for 3 h in the presence of polyols showed an increase in the number of bacteria able to survive (erythritol: *p* = 0.026, *p* = 0.019, *p =* 0.0002), (sorbitol: *p* = 0.036, *p* = 0.034, *p* = 0.002) (Fig. 3a) compared with GBS-S19 incubated for 3 h in the absence of polyols (Fig. 3b). Interestingly, GBS-S19 grown in the presence of 1% mannitol and subsequently incubated with 1% mannitol in blood did not have as significant a survival percentage (*p* = 0.32) as erythritol or sorbitol, however, GBS-S19 did show enhanced survival when grown and then incubated in 2% or 4% mannitol (*p* = 0.036, *p* = 0.035) (Fig. 3a).

We also performed the same assay using lower concentrations of polyols (0.8 to 12 μM) that fall within the physiological concentrations of erythritol in the human conceptus [29, 30]. GBS survival in human blood in the presence of lower concentrations of polyols (0.02, 0.04, 0.06, 0.08, and 0.1%) was not significant (data not shown), suggesting any phenotypic changes seen by GBS only occurs in environments with higher concentrations of polyols.

### Effect of polyols on human white blood cell (hWBC) viability

**Fig. 4:**
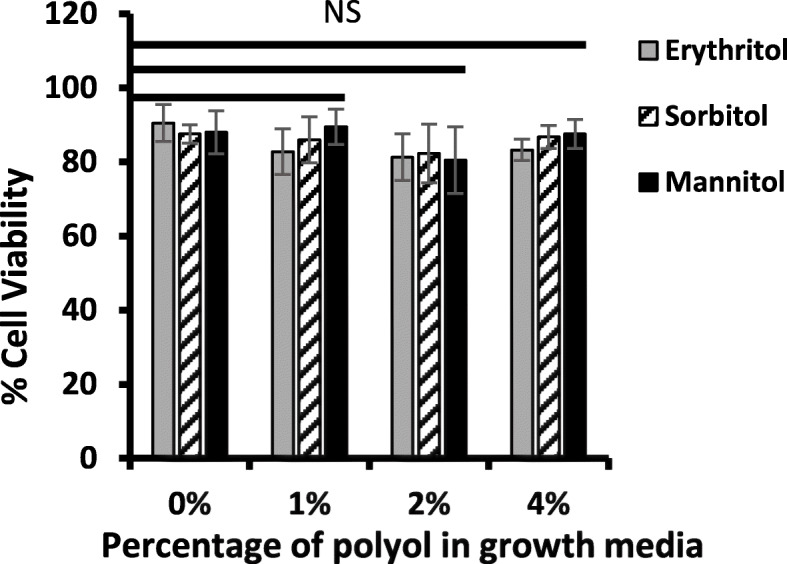
Cell viability assay of hWBCs in the presence of polyols. Trypan blue viability assay showing the percentage of hWBCs viable after 3 h of incubation with 1%, 2% or 4% of polyols. The number of clear (viable) and blue (nonviable) cells was calculated and expressed as percent cell viability. Bars represent the average values obtained from four independent experiments compared with the value obtained from (0%) to determine statistical significance; N.S indicates not significant

To determine if the increased survival of GBS-S19 in fresh human blood in the presence of polyols was due to the ability of the bacteria to survive phagocytic killing or was due to adverse (cytotoxic) effect of polyols on human cells, a cell viability assay was done. A trypan blue dye exclusion test showed that the addition of polyols to human blood had no significant effect on the viability of hWBCs (Fig. 4).

### Invasion of HeLa cells by GBS-S19 in the presence of polyols

**Fig. 5:**
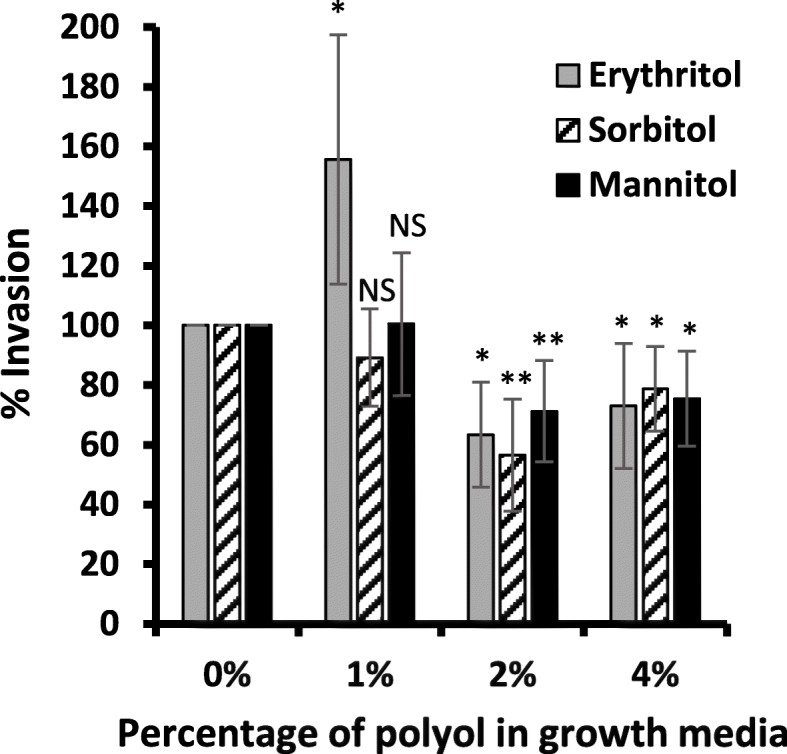
Invasion of HeLa cells by GBS-S19 after growth in 1%, 2% or 4% erythritol, sorbitol, mannitol. Intracellular CFU were recovered after 2 h of infection and were expressed as percent invasion. Internalized GBS grown in 0% polyols were 100-150 CFUs per monolayer. Bars represent the average values obtained from three independent experiments compared with the value obtained from (0%) to determine statistical significance; an asterisk (*) indicates statistical significance (*p* < 0.05), (**) indicates (*p* < 0.01) and NS indicates not significant

GBS can invade a variety of cell types. To understand if the ability of GBS to invade epithelial cells could be altered by polyols, GBS-S19 grown in the presence of erythritol, sorbitol or mannitol was assayed for the bacteria’s ability to invade HeLa cells. Enhanced invasion of HeLa cells was observed for GBS grown in 1% erythritol however the effect was not seen for 1% sorbitol or mannitol (*p =* 0.041, *p =* 0.118, *p =* 0.484) (Fig. 5). In contrast, GBS treated with higher concentrations of polyols (2 and 4%) significantly reduced the number of intracellular CFUs for all three polyols assayed (erythritol: *p =* 0.011, *p =* 0.045), (sorbitol: *p =* 0.002, *p =* 0.012), (mannitol: *p =* 0.007, *p =* 0.011) (Fig. 5).

### Effects of polyols on in vitro GBS-S19 biofilm formation

**Fig. 6:**
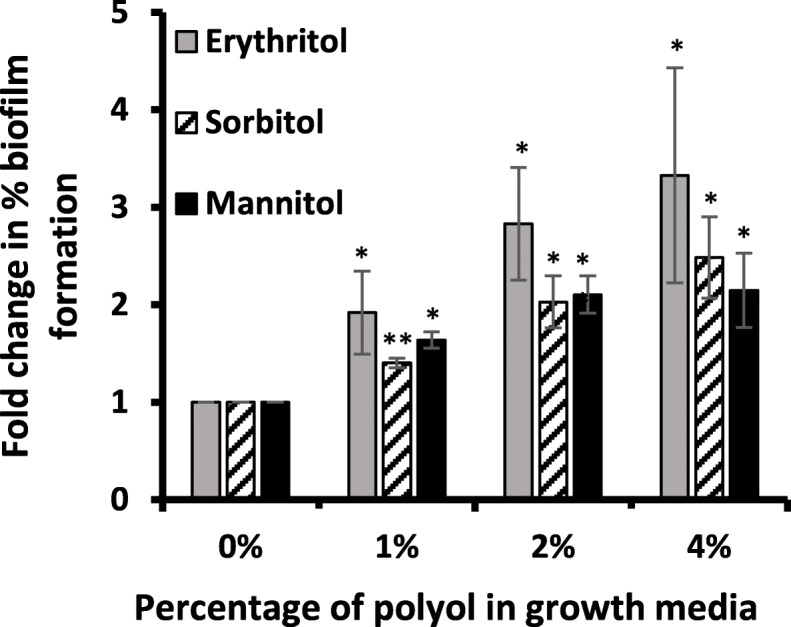
Effects of polyols on GBS-S19 biofilm formation. Biofilm formation of GBS-S19 in TH broth supplemented with polyols was quantified by crystal violet staining. Biofilm formation was expressed as a fold change in the percent biofilm formation in comparison to control (no polyols). Bars represent the average A_595_ values obtained from two independent experiments compared with the value obtained from (0%) to determine statistical significance; an asterisk (*) indicates statistical significance (*p* < 0.05) and (**) indicates (*p* < 0.01)

The effect of polyols on in vitro GBS-S19 biofilm formation was also evaluated. The addition of 1, 2 and 4% of polyols to growth media significantly increased biofilm formation by GBS-S19 (erythritol: 1.9 to 3.3-fold), (sorbitol: 1.4-2.4 fold), (mannitol: 1.6 to 2.15 fold) compared with un-supplemented media (0%) (erythritol: *p* = 0.046, *p* = 0.023 and *p =* 0.048), sorbitol: *p* = 0.004, *p* = 0.016 and *p* = 0.019), mannitol: *p =* 0.004, *p =* 0.007, *p =* 0.025) (Fig. 6).

### Effects of polyols on GBS-S19 hemolytic activity

**Fig. 7:**
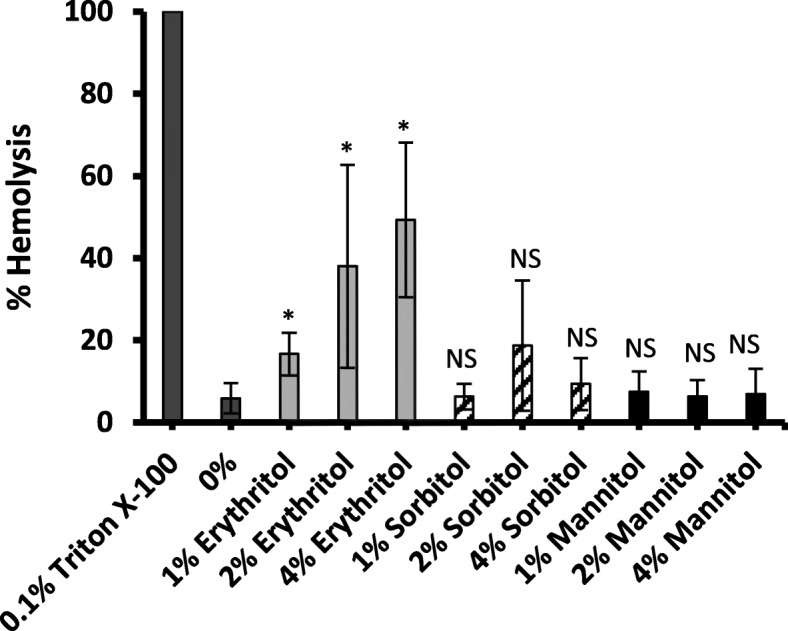
The effect of polyols on GBS-S19 hemolytic activity. Hemolytic activity of GBS-S19 was quantified by measuring the hemolysis of erythrocytes in the plasma of whole human blood treated with polyols minus the hemolysis of erythrocytes in the plasma from whole human blood treated with polyols without GBS-S19 addition. Hemolysis was expressed as percent hemolysis. 0.1% Triton X-100 was considered 100% hemolytic activity. 0% represents the hemolysis observed when GBS was grown in the absence of polyols or glucose. Bars represent the average A_420_ values obtained from two independent experiments compared with the value obtained from (0%) to determine statistical significance; an asterisk (*) indicates statistical significance (*p* < 0.05) and (**) indicates (*p* < 0.01)

GBS are hemolytic bacteria. Hemolysis occurs due to the expression of a ß-hemolysin/cytolysin toxin. To understand if hemolysis activity was affected by the presence of polyols, a hemolytic assay was done with and without polyols. It was found that the addition of erythritol at 1, 2% or 4% to whole human blood significantly increase erythrocyte hemolysis by GBS-S19 compared to GBS-S19 grown without polyols (*p* = 0.035, *p* = 0.043 and *p =* 0.004, respectively) (Fig. 7). However, no significant hemolysis was observed with the same concentrations of sorbitol or mannitol (sorbitol: *p* = 0.33, *p* = 0.17 and *p* = 0.35), (mannitol: *p =* 0.12, *p =* 0.19, *p =* 0.24) (Fig. 7).

### Genome sequence analysis of GBS-S19

The genome sequence analysis of GBS-S19 MLST genes showed this strain to be ST17. To look for genes potentially involved in erythritol metabolism, erythritol catabolism protein sequences from *Brucella* spp., *S. meliloti*, and *R. leguminosarum* were searched in the EggNOG 5.0.0 web interface to identify COG members. Orthologs were found in COG0578 (gbs0264 (GBS strain: PSS_7678)), COG1070 (gbs1844 (GBS strain: NEM316)), and COG1082 (gbs1843 (GBS strain: NEM316)). The GBS-S19 genome contains gbs0264, gbs1843, and gbs1844 at > 98% amino acid identity. Additional GBS orthologs were members of COG0578, COG0170, and COG1082 in EggNOG derived from *Streptococcus hypovaginalis* LMG_14747, however, the taxonomic classification of this genome in the Genome Taxonomic Database (GTDB release 04-RS89) differed from the NCBI taxonomy at the species level (gtdb.ecogenomic.org/genomes?gid=GCF_000323065.2; accessed 2020-06-08) [31, 32].

### Polyol utilization by GBS-S19

**Fig. 8:**
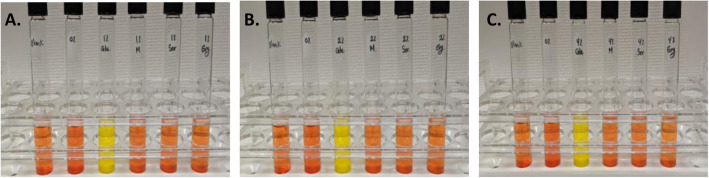
Polyol utilization test by GBS-S19 using tryptone water. Polyols or glucose (**a**) 1%, (**b**) 2% and (C) 4% were added to 10 ml of tryptone water with phenol red and inoculated with GBS-S19. Tryptone water without GBS-S19 inoculation was used as a negative control (Blank). Glucose was used as a positive control and showed acid production (yellow color). Glu. (glucose), Ery. (erythritol), Sor. (sorbitol), M. (mannitol)

**Fig. 9:**
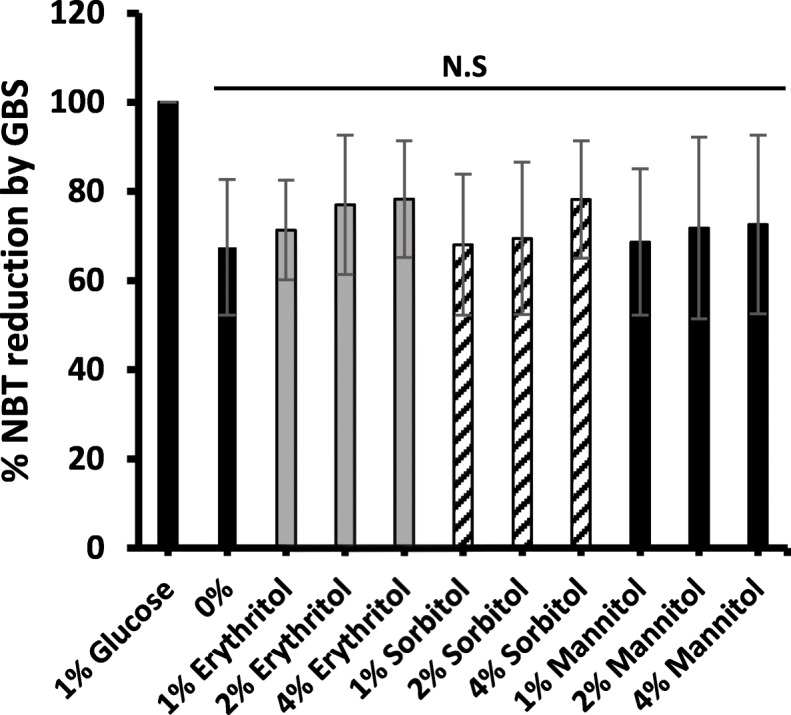
NBT reduction assay by GBS-S19 in the presence of polyols. Glucose or polyols were added to 100 μl of GBS-S19 bacterial suspension and incubated with 0.5 mg/ml of NBT overnight at 37 °C. GBS-S19 incubated with 1% of glucose was used as a positive control. The absorbance A_570_ was expressed as percent of NBT reduction by GBS. 1% of glucose was considered 100% of NBT reduction. Order of tubes for each panel: blank, no polyol, glucose, mannitol, sorbitol, erythritol. Bars represent the average A420 values obtained from three independent experiments compared with the value obtained from (0%) to determine statistical significance; N.S indicates not significant

Genome sequence analysis of GBS-S19 identified orthologous genes that maybe involved in erythritol catabolism. To investigate whether GBS-S19 can utilize erythritol as a carbon source, a polyol utilization test was performed using tryptone water with phenol red. It was shown that varying concentrations of erythritol did not support GBS-S19 growth in tryptone water compared to glucose (Fig. 8). The ability of GBS-S19 to utilize other polyols, sorbitol and mannitol, was also investigated. In addition to erythritol, sorbitol and mannitol did not support GBS-S19 growth (Fig. 8). To further determine if GBS could utilize and oxidize polyols, a nitroblue tetrazolium (NBT) reduction assay was done (Fig. 9). GBS was unable to reduce NBT when grown in the presence of polyols indicating the assayed polyols are not used as a carbon source.

## Discussion

Various polyols or sugar alcohols have been found to alter the phenotype of different bacteria such as *Brucella abortus* and *Burkholderia multivorans* affecting their ability to cause disease [20, 25]. We wanted to determine if polyols could also cause phenotypic changes in the invasive pathogen, GBS. We first selected a strain of GBS that was an invasive clinical isolate and not laboratory adapted. Past studies of *Brucella* have suggested long passages during laboratory culture may decrease erythritol utilization and virulence [20]. To avoid this potential effect, we selected a highly pathogenic GBS strain from a case of severe neonatal invasive neurologic disease that had experienced limited laboratory passages [33].

We found no significant inhibition (or enhancement) of growth of GBS when the bacteria were grown in the presence of polyols assayed. This contrasted with studies of two oral streptococcal species (*Streptococcus mutans* and *Streptococcus gordoni*) in which it was shown that these bacteria can be inhibited to varying degrees when cultured in media containing small percentages of erythritol or xylitol [15, 34]. A similar observation to the differences in growth between oral streptococci and GBS in the presence of polyols was also observed with biofilm formation. In the presence of specific concentrations of polyols, biofilm formation of oral streptococci was previously shown to decrease, whereas an increase in biofilm formation was observed in our study with GBS [13–16].

A possible explanation for these differences between GBS VS oral streptococci may be related to environmental niches each occupies. *S. mutans* and *S. gordonii* are oral streptococci whereas GBS typically inhabit the urogenital tract, both different environments for which the bacteria likely have adapted to allow growth [15, 35]. However, it is also possible that different species of bacteria may respond differently to environmental changes in terms of regulating metabolic pathways that can lead to biofilm formation.

GBS express several surface and secreted proteins of which one is GBS-PGK [36–39]. We have previously shown that GBS can secrete GBS-PGK which can then bind back to the GBS cell surface and subsequently bind plasminogen [28]. The observation that GBS grown in the presence of erythritol significantly increased PGK surface expression suggests erythritol can trigger upregulation of the surface expression of the GBS-PGK. A similar effect has also been observed in Brucella in which expression of PGK is increased in the presence of erythritol [20, 26].

The finding that erythritol can increase surface expression of PGK, prompted us to determine if erythritol also had any effect on the ability of GBS to survive in fresh human blood in the presence of polyols (anti-phagocytic activity). The increase in the numbers of GBS incubated in human blood in the presence of erythritol showed that GBS was able to prevent phagocytic killing when exposed to erythritol. How erythritol may cause this effect in GBS is unclear. Petersen et al. previously demonstrated that *Brucella* growth in erythritol resulted in a significant upregulation of genes associated with the VirB secretion system of *Brucella* [20]. The VirB system regulates a collection of *Brucella* genes which are essential for intracellular growth in cell culture [20]. An analogous system in GBS would provide a plausible explanation for increased GBS survival. It is known that many proteins associated with GBS virulence are regulated by the two-component regulatory CovR/S system. Cumley et al. have previously demonstrated that loss of CovR/S in GBS prevents intracellular survival within macrophages indicating this regulator is important for intracellular survival [40]. While erythritol alone does not seem to influence GBS growth, this polyol may still have an effect on the CovR/S system upregulating expression of proteins promoting the bacteria’s intracellular survival.

The ability of erythritol to alter the phenotype of GBS was further shown when 1% erythritol significantly increased GBS invasion of HeLa cells. Further evidence that erythritol alters the GBS phenotype was the observation of this polyols ability to increase hemolysis of erythrocytes as well as increase biofilm formation, both characteristics associated with GBS virulence. GBS hemolysis is caused by a β-hemolysin/cytolysin, a pore forming toxin that can promote host cell invasion [41–45]. Biofilm formation is associated with bacterial persistence [46]. Both activities are tightly regulated by GBS. Our results suggest erythritol is either directly or indirectly influences this regulation in GBS leading to these phenotypic changes.

Previous studies have shown that erythritol modified the virulence of Brucella, Chlamydia and oral streptococci prompting similar experiments to be done in GBS [13–16, 19–24, 27]. Once it was established that erythritol influenced GBS virulence, a limited number of subsequent polyols (sorbitol and mannitol) were assayed. While our studies were limited to these three polyols, the effect of other polyols such as inositol, ribitol and xylitol should be explored in future studies.

It is interesting to note that while GBS growth was not inhibited, its growth was not enhanced either in the presence of polyols. Other bacteria such as *Brucella* show a strong preference for growth in the presence of erythritol [22, 23]. This is accomplished through the activation of a specific set of genes that catabolize erythritol (*ery* operon) in *Brucella* [24, 47]*.* A search for amino acid sequences similar to *eryABC* in *Brucella* identified COGs containing similar sequences in two GBS strains, PSS_7678 and NEM316, both serotype III strains [48]. The GBS-S19 genome contained three similar genes (gbs0264, gbs1843 and gbs1844). These genes provide the possibility that GBS-S19 may potentially catabolize erythritol, however, the lack of growth of GBS-S19 in media containing this polyol suggests these proteins either are not involved in the promotion of growth of the bacteria or erythritol cannot be exclusively used as a carbon source. Further work involving these proteins is necessary to determine if they play roles in promoting the phenotypic changes we have described.

The effect of erythritol on GBS virulence was observed with concentration at 1% (82 μM). While the concentrations of polyols used in the experiments described are relatively high compared to the physiological concentrations of polyols in the human conceptus, the changes in phenotypic characteristics upon polyol exposure are new. High concentrations of polyols maybe a limitation to our study, however, the concentrations used were selected based on previous studies that explored the effects of polyols on other streptococcal species and brucella [15, 19, 20].

## Conclusions

Polyols such as erythritol, increased expression of surface PGK, enhanced the bacteria’s ability to survive in an anti-phagocytic environment, increased bacterial hemolytic activity, increased GBS biofilm formation, and at low concentrations, more efficiently invaded HeLa cells. Together these phenotypic changes suggest exposure to polyols such as erythritol can potentially enhance the virulence of the bacteria. Polyols such as erythritol, inositol, mannitol and sorbitol are present in human trophoblastic cells, coelomic and amniotic fluid an environment where GBS are known to cause disease [27, 29, 30]. It is possible that exposure of GBS to polyols such as erythritol at these sites may potentially alter the phenotype of GBS leading to a change in various virulence properties.

## Methods

### Bacterial strain, cell line, and growth conditions

A previously described GBS serotype III isolate collected from a case of invasive GBS disease was used in this study [33]. This strain was designated GBS-S19. GBS-S19 was grown in 2 ml of Todd-Hewitt (TH) broth overnight at 37 °C and then transferred to 5 ml of TH supplemented with varying concentrations of polyols. The supplemented TH broth contained 1% (0.08 mol/L), 2% (0.16 mol/L) or 4% (0.32 mol/L) of erythritol (NowFood, USA), or 1% (0.055 mol/L), 2% (0.11 mol/L) or 4% (0.22 mol/L) of sorbitol (Sigma-Aldrich, St. Louis, USA), or 1% (0.055 mol/L), 2% (0.11 mol/L) or 4% (0.22 mol/L) of mannitol (Sigma-Aldrich, St. Louis, USA). The concentrations of polyols used was similar to concentrations of erythritol used in previous studies reporting erythritol’s effect on Brucella and oral streptococci [15, 19, 20]. Stocks of the polyols were prepared as 50% concentrations of each polyol by dilution in sterile distilled water and sterilized by filter sterilization using 0.22 μm sterile syringe filters (Millipore Sigma, Oakville, Ontario, Canada). Dilutions were done with TH broth to achieve the desired concentration of polyol. Controls consisted of TH broth only. Bacterial cells were cultured in a shaker incubator at 37 °C. Growth was monitored by measuring the absorbance spectrophotometrically at a wavelength of 600 nm. Time points for measuring were 0, 2, 4, 6, 8 and 24 h.

The human epithelial cell line HeLa 229 (ATCC CCL-2.1) (ATCC, Manassas, VA, USA) was grown in OPTI-MEM I reduced serum medium supplemented with 4% fetal bovine serum (FBS) (Thermo Fisher Scientific, Toronto, Canada).

### Assay for GBS-PGK surface expression in GBS-S19

The expression of GBS-PGK on the surface of GBS-S19 was assayed using an ELISA as previously described with modifications [49]. Five ml of GBS-S19 was grown overnight in TH broth supplemented with polyols. A 5 ml growth control with no polyols added was also used. This was then centrifuged to pellet the bacteria and washed once with 1 ml of 1X Tris-buffered saline (pH 7.0) (TBS) and re-suspended in 1 ml of TBS. One hundred microliters of washed bacterial cells were added to the wells of a 96-well polystyrene plate (Maxi-sorp; NUNC, Thermo Fischer Scientific, Nepean, Canada) and incubated for 2 h at 37 °C to allow bacterial cells to adhere to the plastic. Wells were washed once with TBS and blocked with 5% skim milk in TBS for 1 h. After blocking, the wells were washed 3 times with TBS and incubated for 1 h with anti-rGBS-PGK antibodies raised in rabbits (1:300 diluted in blocking buffer) [28]. After 1 h, wells were further washed 3 times with TBS and incubated 1 h with anti-rabbit IgG-Horse-Radish Peroxidase conjugated antibodies (Sigma-Aldrich) (1:1000 diluted in blocking buffer). After 1 h, wells were washed 3 times with TBS and developed with 50 μl of 3,3′,5,5′-Tetramethylbenzidine (TMP) (Sigma-Aldrich, St. Louis, USA) for 30 min at room temperature (RT) before stopping the reaction with 50 μL of 2 M sulphuric acid (H_2_SO_4_). The absorbance at 450 nm (A_450_) was measured using an Athos LP400 microplate reader (Bio-Rad Laboratories Ltd., Mississauga, Canada). The A_450_ values obtained were compared with the average A_450_ measurement from GBS-S19 bacterial cells grown without polyols (0%).

### Anti-phagocytic activity of GBS grown in polyols

To determine the ability of GBS-S19 grown in polyols to resist phagocytosis and survive in heparinized human blood, the assay was performed as previously described with minor modifications [50]. Overnight 5 ml cultures of GBS-S19 were grown in TH broth supplemented with polyols. After growth, the cultures were centrifuged to pellet the bacteria and washed once with 1X TBS buffer. A 0.5 McFarland standard of the bacterial culture was made and diluted in TH broth (1:10,000) to obtain 1.5 X 10^3^ CFU/ml for the initial inoculum of the assay. Fifty microliters of the suspension, containing approximately 75 CFU, was added to 250 μl of freshly heparinized blood in sterile test tubes either with or without addition of polyols, and incubated at 37 °C under gentle rotation. After 3 h of incubation, 150 μl aliquots of each of the suspensions were plated on blood agar plates (BAP) (Dalyn Biologicals, Calgary, Canada) to determine the number of CFU after growth in human blood. BAPs were incubated overnight at 37 °C. Growth of GBS-S19 in human blood was expressed by calculating the number of CFU obtained at the end of the incubation period over the number of CFU obtained at time zero (T_0_). The growth of GBS in TH broth without polyol supplementation was used as a negative control for anti-phagocytic activity. Blood was collected from healthy volunteers in 6 ml Sodium Heparinized Vacutainer tubes (BD, Oakville, Canada).

### Cell viability assay

The effect of polyols on cell viability of hWBCs was assessed using a trypan blue exclusion method. Aliquots of human blood were incubated with polyols for 3 h at 37 °C under gentle rotation. After 3 h incubation and before counting the WBCs, red blood cells (RBCs) were removed. RBCs were lysed using 3X volume of distilled water for 30-60 s, followed by addition of 3X volume of 1X PBS to recover the isotonic condition. Following lysis of RBCs, the mixture was centrifuged for 3 min at 3000 x g and pellets resuspended in 50 μl of 1X PBS. Equal parts of cell suspension and 0.4% trypan blue dye were mixed by pipet followed by loading 10 μl of the mixture into a hemocytometer counter to count all clear (live) and blue (dead) cells. Cell viability was calculated by dividing the number of viable cells by the number of total cells and multiplying by 100%.

### Invasion assay of HeLa cells by GBS grown in the presence of polyols

To determine the effect of polyols on the invasion by GBS into a cultured cell line, a standard antibiotic protection assay was performed as previously described using HeLa cells [51]. HeLa cell culture monolayers were grown to confluence in 24 well plates (Corning Costar TC-Treated multiple well plates, Sigma-Aldrich, St. Louis, USA). Five ml cultures of GBS-S19 were grown overnight in TH broth supplemented with varying concentrations of polyols and washed once with 1X TBS. A 0.5 McFarland standard of each culture was made and then diluted 1:100 in TH broth. Immediately, 100 μl (1.2 X 10^5^ CFU) of 1:100 dilutions (giving a multiplicity of infection of 1:1) were added to the monolayer. The plates were centrifuged at 100 x *g* for 5 min and incubated at 37 °C in 5% CO_2_ for 2 h. The HeLa cell monolayer was then washed 3 times with PBS and incubated for 2 h with fresh cell culture media containing 5 μg/ml of penicillin and 100 μg/ml of gentamicin to kill any remaining extracellular bacteria. The HeLa cell monolayers were washed again with PBS, treated with trypsin, lysed with 0.1% Triton X-100, and plated on TH agar for quantitation of intracellular CFUs. Percent invasion was calculated as: Number of CFU (GBS treated with polyols) invaded into HeLa cells/ Number of CFU (GBS untreated with polyols) invaded into HeLa cells X 100.

### Biofilm formation assay

Five ml cultures of GBS-S19 grown overnight at 37 °C in TH broth was diluted 1:20 in fresh TH broth supplemented with polyols. As a control, 1:20 culture dilution was made in fresh TH broth without polyols. Two hundred microliters of each of the mixtures was aliquoted into 96-well plates. Three wells were used for each sample. Cells were grown under static conditions at 37 °C for 20 h. Following incubation, unattached bacteria were removed by washing twice with 1X PBS (200 μl), and attached bacteria were stained with 100 μl 1% crystal violet for 10 min at RT. Wells were washed three times with 200 μl PBS and the bound crystal violet was solubilized with 200 μl of 95% ethanol with gentle agitation for 20 min at RT. The absorbance of the eluents was measured at 595 nm. All assays were repeated two times in triplicate. The A_595_ values obtained were compared with the average A_595_ measurement from GBS-S19 bacterial cells grown without polyols (0%). Biofilm formation was expressed as percent biofilm formation.

### Effect of polyols on GBS hemolysis

GBS-S19 was grown in 5 ml at 37 °C overnight and a 0.5 McFarland standard made from this culture. Ten microlitres was added to 0.5 m l of freshly heparinized whole human blood with added polyols and incubated at 37 °C under gentle rotation. Control consisted of polyols added to 0.5 m l of whole human blood at 1, 2% or 4% concentrations without addition of GBS-S19 and incubated at 37 °C under gentle rotation. After 3 h of incubation, tubes were centrifuged for 3 min at 3000 x g and 100 μl plasma from each tube (duplicate) was transferred to 96 well plates. The plasma from whole human blood treated with 0.1% Triton X-100 was used as a positive control. The absorbance was measured at 420 nm. GBS-S19 hemolytic activity was calculated by measuring the A420 of plasma from whole human blood treated with polyols and incubated with GBS minus the A420 of plasma from whole human blood treated with polyols only. The obtained value was converted to percentage compared to the A420 value obtained from 0% and presented as percent hemolysis.

### Genome analysis of GBS-S19

DNA from a single colony of GBS-S19 was extracted using MagnaZoreb (Promega) and 350 ng was used as input for library preparation with Illumina Nextera DNA Flex kit, before sequencing on an Illumina MiSeq with a 600 cycle V3 sequencing kit. Raw sequence quality was assessed with FastQC v0.11.9 (www.bioinformatics.babraham.ac.uk/projects/fastqc/) and a draft genome was assembled using Shovill 1.1.0 (github.com/tseemann/shovill) and SPAdes 3.14.0 [52]. Genome assembly quality was assessed with QUAST v5.0.2 [53]. Multi-locus sequence typing was done in silico using mlst 2.19.0 (github.com/tseemann/mlst) and the PubMLST database [54]. Known erythritol catabolic protein sequences from *Brucella* spp. (WP_002972113.1 (carbohydrate kinase; *eryA*), WP_002965781.1 (glycerol-3-phosphate dehydrogenase; *eryB*), WP_002965780.1 (aminotransferase; *eryC*)), *Sinorhizobium meliloti* (CAC46814.1, CAC46811.1, CAC46807.1), and *Rhizobium leguminosarum* (CAK11916.1, CAK11917.1, CAK11918.1) were used to identify Cluster of Orthologous Groups (COG) that included orthologs from *S. agalactiae* using EggNOG 5.0.0 [55]. Confirmation of *S. agalactiae* erythritol catabolic protein sequences in the GBS-S19 genome was confirmed using tblastn 2.9.0 [56].

### GBS utilization of polyols

Tryptone water (10 g/L tryptone, 5 g/L NaCl, (pH 7.0), and 7.2 ml of 0.25% phenol red) was first prepared and then supplemented with polyols at the required concentrations. GBS-S19 was added to the media and incubated overnight at 37 °C. The ability of GBS-S19 to catabolize polyols was determined by a colour change of the media to yellow, which indicates pyruvic acid production. Tryptone water uninoculated with GBS-S19 was used as a negative control. A positive control consisted of glucose (1, 2%, or 4%) in tryptone water.

### Nitroblue tetrazolium reduction assay

Five ml cultures of GBS-S19 were grown overnight at 37 °C in TH broth. After growth, the cultures were centrifuged to pellet the bacteria and washed once with PBS and re-suspended in 5 ml of PBS. One hundred μl of cell suspension was aliquoted into Eppendorf tubes with addition of polyols at the required concentrations. In this assay, 1% of glucose was added to the cell suspension as a positive control. In all the tubes, 0.5 mg/ml of NBT (Sigma) was added and incubated overnight at 37 °C. After incubation, 900 μl of PBS were added to each tube, and the absorbance was measured at 570 nm. The A570 values obtained were compared with the average A570 measurement from GBS-S19 bacterial cells incubated without polyols.

### Statistical analysis

Data was analyzed using the Students t test and a *p*-value < 0.05 was considered statistically significant. Data points correspond to the average value of all replicates and error bars represent the standard deviation.

## Data Availability

The datasets used/or analyzed during the current study are the figures included in this published article. The genome sequence data for GBS-S19 is available in the GenBank repository under BioProject PRJNA642879. Website: www.ncbi.nlm.nih.gov/nuccore/?term=SAMN15400523

## Abbreviations

A: Absorbance
°C: Celsius
BAP: Blood agar plates
CFU: Colony forming unit
FBS: Fetal bovine serum
GBS: Group B Streptococcus
h: hour
Min: Minutes
PGK: Phosphoglycerate kinase
PBS: Phosphate buffer saline
RT: Room temperature
TBS: Tris-buffered saline
TH: Todd-Hewitt
TMP: 3,3′,5,5′-Tetramethylbenzidine
